# Evaluation of the GenoType NTM-DR assay performance for the identification and molecular detection of antibiotic resistance in *Mycobacterium abscessus* complex

**DOI:** 10.1371/journal.pone.0239146

**Published:** 2020-09-25

**Authors:** Nicolas Bouzinbi, Olivier Marcy, Thibault Bertolotti, Raphael Chiron, Pascale Bemer, Martine Pestel-Caron, Olivia Peuchant, Hélène Guet-Revillet, Marie-Sarah Fangous, Geneviève Héry-Arnaud, Abdoul-Salam Ouedraogo, Anne-Laure Bañuls, Sylvain Godreuil

**Affiliations:** 1 Laboratoire de Bactériologie, Centre Hospitalier Universitaire de Montpellier, Montpellier, France; 2 MIVEGEC, IRD, CNRS, Université de Montpellier, Montpellier, France; 3 Bordeaux Population Health Centre U1219, Université de Bordeaux, Bordeaux, France; 4 Cystic Fibrosis Centre, University Hospital of Montpellier, Montpellier, France; 5 Bacteriology-Hospital Hygiene Department, University Hospital of Nantes, Nantes University, Nantes, France; 6 UNIROUEN, GRAM EA2656, Rouen University Hospital, Normandie Université, Rouen, France; 7 USC EA 3671 Infections Humaines à Mycoplasmes et à Chlamydiae, Univ. Bordeaux, Bordeaux, France; 8 CHU de Toulouse, Laboratoire de Bactériologie-Hygiène, Institut Fédératif de Biologie, Toulouse, France; 9 Bacteriology-Hospital Hygiene Department, University Hospital of Brest, Brest University, Brest, France; 10 Laboratory Department, Sourô Sanou University Hospital, Bobo-Dioulasso, Burkina Faso; 11 LMI Drug Resistance in South East Asia “DRISA”, IRD Montpellier, Montpellier, France; Nitte University, INDIA

## Abstract

The first objective of this study was to determine the GenoType NTM-DR assay performance for subspecies identification in *Mycobacterium abscessus* complex isolates. The second objective was to evaluate the GenoType NTM-DR assay ability to detect clarithromycin and amikacin resistance in *M*. *abscessus* complex isolates compared with drug susceptibility testing (DST) and PCR sequencing of the *erm(41)*, *rrl* and *rrs* genes. The concordance between the GenoType NTM-DR and MLST results concerning subspecies identification was 100%. The wild type and mutated alleles of the *rrl* and *rrs* genes were detected by the GenoType NTM-DR assay and PCR sequencing with 100% (115/115) agreement. Similarly, 100% concordance between GenoType NTM-DR and DST was observed for clarithromycin and amikacin testing. Sensitivity for the detection of clarithromycin and amikacin resistance was 100%. The GenoType NTM-DR assay provides a robust and complementary tool to the gold standard methods (MLST and broth microdilution) for subspecies identification and drug resistance detection.

## Introduction

The fast-growing *Mycobacterium abscessus* complex is an emerging opportunistic human pathogen worldwide [1]. It includes three subspecies: *M*. *abscessus* subspecies *abscessus*, *M*. *abscessus* subsp. *massiliense*, and *M*. *abscessus* subsp. *bolletii* [2]. The most frequent clinical manifestation of *M*. *abscessus* complex infection is chronic lung infection, usually in elderly women with bronchiectasis and in young adults with cystic fibrosis (CF) [3].

*M*. *abscessus* carries or acquires inducible resistance to macrolides (e.g. clarithromycin) and aminoglycosides (e.g. amikacin) used to treat pulmonary infections. Resistance to macrolides can be: i) constitutive (associated with point mutations at position A2058 and A2059 of the *rrl* gene, a macrolide target), in all subspecies; and ii) inducible (associated with the *erm(41)* gene T28 sequevar), only in *M*. *abscessus* subsp. *abscessus* and *bolletii* [4]. A single 16S ribosomal RNA (rRNA) substitution at position 1408 in the *rrs* gene causes high-level aminoglycoside resistance [4, 5]. Rapid molecular diagnosis is essential for subspecies identification and determination of the adequate antimicrobial therapy, and is now recommended in patients with CF [3, 6].

The recently commercialized GenoType NTM-DR test (NTM-DR) (Hain, Lifescience, Nehren, Germany) allows the rapid *M*. *abscessus* subspecies identification and simultaneously the detection of resistance to macrolides and aminoglycosides [7–9].

This study wanted to 1) determine NTM-DR performance for subspecies identification in *M*. *abscessus* complex isolates (compared with MLST), and 2) evaluate NTM-DR ability to detect clarithromycin and amikacin resistance (compared with phenotypic and sequencing analyses).

## Materials and methods

### Patients and mycobacteria isolates

For this study, 115 *M*. *abscessus* complex isolates (1 isolate/patient) were randomly selected among the 176 isolates (1 isolate/patient) from respiratory samples of 176 different patients with CF or other respiratory diseases stored at the Microbiology Laboratory, University Hospital, Montpellier (France). All *M*. *abscessus* complex isolates came from six University Hospitals in France (Brest, Bordeaux, Montpellier, Nantes, Rouen, and Toulouse), including a Reference Centre for CF.

### Molecular analysis and drug susceptibility testing

Total DNA was extracted using GenoLyse v2.0 (Hain Lifescience, Nehren, Germany). Isolates were assigned to the *M*. *abscessus* complex using the multiplex GenoType Mycobacterium CM assay (Hain Lifescience Nehren, Germany), and subspecies were identified with the GenoType NTM-DR Kit (Hain Lifescience, Nehren, Germany), the index test, according to the manufacturer’s recommendations. All isolates were also characterized by multilocus sequence typing (MLST), based on eight housekeeping genes (*argH*, *cya*, *glpK*, *gnd*, *murC*, *pgm*, *pta*, and *purH*), as previously described [10]. *M*. *abscessus* complex subspecies, allele and sequence type were identified using the *M*. *abscessus* MLST database (http://www.pasteur.fr/recherche/genopole/PF8/mlst/Myco-abscessus).

Resistance was assessed using the GenoType NTM-DR Kit and by phenotypic drug susceptibility testing (DST), the reference method [11]. The Minimum Inhibitory Concentrations (MICs) of clarithromycin and amikacin were evaluated using the reference broth microdilution method with Sensititre RAPMYCO microplates (Trek Diagnosis Systems). Susceptibility and resistance were assessed according to the CLSI recommendations. High-level clarithromycin resistance was defined by a MIC ≥8 μg/ml at day 5. Inducible resistance was defined by an increase in clarithromycin MIC from ≤2 μg/ml at day 5 to ≥8 μg/ml at day 14. Aminoglycoside resistance was defined by MIC values ≥64 μg/ml [11]. Mutations in the *erm(41)*, *rrl* and *rrs* genes were identified by PCR and sequencing, as previously described [12, 13].

NTM-DR detects heteroresistance in mixed populations of drug-susceptible and -resistant mycobacteria and shows the simultaneously the presence of wild type (WT) and mutant (MUT) bands.

### Statistical analyses

Based on an expected NTM-DR assay sensitivity of 96% for subspecies identification (100% and 92% according to the manufacturer and Kehrmann et al. [7], respectively), 115 of the 176 isolates (60 *M*. *abscessus* subsp. *abscessus*, 47 *M*. *M*. *abscessus* subsp. *massiliense*, and 8 *M*. *abscessus* subsp. *bolletii*) were needed to assess the test sensitivity with a precision of ±5%.

## Results

The agreement between NTM-DR and MLST results for subspecies identification was 100% (60/60) for *M*. *abscessus* subsp. *abscessus*, 100% (47/47) for. *M*. *abscessus* subsp. *massiliense*, and 100% (8/8) for *M*. *abscessus* subsp. *bolletii*. NTM-DR sensitivity and specificity for *M*. *abscessus* subsp. *abscessus* identification were 100% [95% CI: 96.2–100] and 100% [95% CI: 93.5–100].

Both NTM-DR and DST identified 59/115 (51.3%) isolates as resistant to clarithromycin: 12 (n = 7 *M*. *abscessus* subsp. *abscessus* and n = 5 *M*. *abscessus* subsp. *massiliense*) with high resistance level and 47 (n = 39 *M*. *abscessus* subsp. *abscessus* and n = 8 *M*. *abscessus* subsp. *bolletii*) with inducible resistance (S1 Table). Both techniques identified six (5.2%, 6/115) isolates resistant to aminoglycosides (n = 4 *M*. *abscessus* subsp. *abscessus* and n = 2 *M*. *abscessus* subsp. *massiliense*) (S1 Table). Agreement was total between NTM-DR and DST results for clarithromycin and aminoglycoside resistance.

NTM-DR identified *rrl* mutations associated with high level of resistance in the 12 isolates with high resistance to clarithromycin (n = 4 A2058C, n = 5 A2058G, and n = 3 A2059G *rrl* mutation, corresponding to the positive MUT1, MTU2 and MUT4 bands of NTM-DR, respectively, (S1 Table). Among the 12 clarithromycin-resistant *M*. *abscessus* subsp. *abscessus* isolates, 11 showed a *rrl* profile without WT band and one presented a heterogeneous pattern (one WT and one MUT1 band), suggesting the presence of a mixed population. In all 47 isolates with inducible clarithromycin resistance, NTM-DR detected a specific *erm (41)* T28 sequevar band and a WT band in the *rrl* gene. Finally, all 56 isolates susceptible to clarithromycin by DST were susceptible also by NTM-DR (*erm(41)* C28 sequevar band and WT band in the *rrl* gene) (S1 Table).

The six isolates with aminoglycoside resistance displayed the positive MUT1 band by NTM-DR. Sequencing confirmed the presence of the A1408G *rrs* mutation in these isolates. The 109 (94.8%) aminoglycoside-susceptible isolates showed a WT band by NTM-DR and WT *rrs* gene sequence (S1 Table). Overall, NTM-DR and sequencing showed 100% agreement. The concordance between NTM-DR and DST was 100% for clarithromycin and amikacin. NTM-DR sensitivity for the detection of clarithromycin and amikacin resistance was also 100%.

## Discussion

Our study showed an excellent concordance (100%) between NTM-DR and MLST results concerning subspecies identification, and confirmed the high sensitivity of NTM-DR for detecting acquired resistance. This is higher than what reported by a recent study on 50 M. *abscessus* complex isolates (92% concordance between NTM-DR and gene sequencing for subspecies identification) [7, 14, 15]. The excellent results observed confirmed that the NTM-DR assay seems a discriminative method for *M*. *abscessus* complex subspecies identification [7, 9, 16].

Our study showed that NTM-DR can detect acquired resistance with high sensitivity. This test also easily detects heteroresistance, not always possible by sequencing [8]. This is crucial for treatment decision-making, and it would be relevant to evaluate *in vitro* NTM-DR sensitivity for heteroresistance detection in mixed populations of drug-susceptible and -resistant mycobacteria.

Although this is not the case for our sample, it has been reported that some isolates with phenotypic resistance to macrolides and aminoglycoside do not carry mutations in the *rrl* and *rrs* genes. This implies the existence of other resistance mechanisms that cannot be detected by the NTM-DR and other commercial kits [7, 8]. For example, a modification (direct repeat 18-bp insertion in rpIV) in the ribosomal protein L4 has been recently associated with resistance to macrolides [17].

## Conclusion

The excellent NTM-DR performance indicates that this test is a robust and complementary tool to MLST and broth microdilution for subspecies identification and detection of clarithromycin and amikacin resistance in *M*. *abscessus* complex. NTM-DR could be used for routine testing, particularly in patients with and without CF and *M*. *abscessus complex* lung infections.

## Supporting information

S1 Table (XLSX)
